# Cross-Sectional Transcriptional Analysis of the Aging Murine Heart

**DOI:** 10.3389/fmolb.2020.565530

**Published:** 2020-09-25

**Authors:** Matthew Greenig, Andrew Melville, Derek Huntley, Mark Isalan, Michal Mielcarek

**Affiliations:** ^1^Department of Life Sciences, Imperial College London, London, United Kingdom; ^2^Department of Mathematics, Imperial College London, London, United Kingdom; ^3^Imperial College Center for Synthetic Biology, Imperial College London, London, United Kingdom

**Keywords:** RNAseq, transcriptomics, heart, aging, cardiomyocyte, gene expression, genetics

## Abstract

Cardiovascular disease accounts for millions of deaths each year and is currently the leading cause of mortality worldwide. The aging process is clearly linked to cardiovascular disease, however, the exact relationship between aging and heart function is not fully understood. Furthermore, a holistic view of cardiac aging, linking features of early life development to changes observed in old age, has not been synthesized. Here, we re-purpose RNA-sequencing data previously-collected by our group, investigating gene expression differences between wild-type mice of different age groups that represent key developmental milestones in the murine lifespan. DESeq2's generalized linear model was applied with two hypothesis testing approaches to identify differentially-expressed (DE) genes, both between pairs of age groups and across mice of all ages. Pairwise comparisons identified genes associated with specific age transitions, while comparisons across all age groups identified a large set of genes associated with the aging process more broadly. An unsupervised machine learning approach was then applied to extract common expression patterns from this set of age-associated genes. Sets of genes with both linear and non-linear expression trajectories were identified, suggesting that aging not only involves the activation of gene expression programs unique to different age groups, but also the re-activation of gene expression programs from earlier ages. Overall, we present a comprehensive transcriptomic analysis of cardiac gene expression patterns across the entirety of the murine lifespan.

## Introduction

As the longevity of the human population increases, aging will continue to present one of the most significant risk factors for cardiovascular disease (Go et al., 2014). Epidemiological models have suggested that by 2035, one in four people will be above 65 years of age (Lakatta and Sollott, 2002), and it is well established that the prevalence of cardiovascular diseases increases considerably with age (Lakatta, 2015). The aging heart undergoes a number of structural and functional changes that significantly increase the risk of heart failure. Typically, age-associated functional changes in the heart manifest as a decrease in left ventricular diastolic function (Desai and Fang, 2008; Keller and Howlett, 2016), reduced systolic function (due to a reduction in cardiac reserve during exercise) (Lakatta and Levy, 2003), and altered electrical functions (Jones, 2006; Strait and Lakatta, 2012). These changes are likely caused by alterations in ventricular and atrial structure; for example, an increase in the left ventricle thickness due to cardiomyocytes hypertrophy (Cheng et al., 2009), or atrial hypertrophy and dilatation (Lam et al., 2017). These pathological changes in the aging heart's structure and function accompany the progression of various diseases, including cardiac hypertrophy and dilated cardiomyopathy (Lakatta, 2015). Interestingly, similar pathological processes within the cardiovascular system have been observed in neurodegenerative disorders (Critchley et al., 2018), including Huntington's disease-related cardiomyopathy, previously identified by our group and others (Mielcarek et al., 2014; Toczek et al., 2016).

Cardiac aging is widely studied in rodents, especially mice, which recapitulate phenotypes of the aging human heart (Dai and Rabinovitch, 2009). Aging mice display similar alterations to those observed in aging humans, particularly changes in left ventricular mass and atrial dimension (Barger et al., 2008), as well as changes in interstitial fibrosis, collapse of sarcomeres, mineralization (calcification), hypertrophic myocyte fiber size, increased cardiomyocytes apoptosis, and amyloid deposition, as reviewed by Dai and Rabinovitch (2009). More importantly, aging mice do not develop increased blood pressure or altered lipid and glucose levels (Zheng et al., 2003; Dai et al., 2009), which makes it possible to study age-related changes in the heart in the absence of pathologies like diabetes and hypertension. While studying the aging process in mice, it is vital to consider that different genetic backgrounds might be associated with distinct aging properties (Kiper et al., 2013). Nevertheless, over the past few decades, murine models have been extensively used to unravel the mechanisms of human cardiac aging, aiming to develop new interventions to attenuate or reverse its progression.

Transcriptome-wide profiling presents an attractive method to study virtually any biological process, including aging. It offers an unbiased way to produce mechanistic insights and serves as an effective method for identifying novel sets of biomarkers. In this study, we surveyed whole-heart transcriptomic variation between four different age groups of mice, aiming to characterize how gene expression in the heart changes throughout the murine lifespan.

## Results

### Differential Expression Analysis

**Figure 1:**
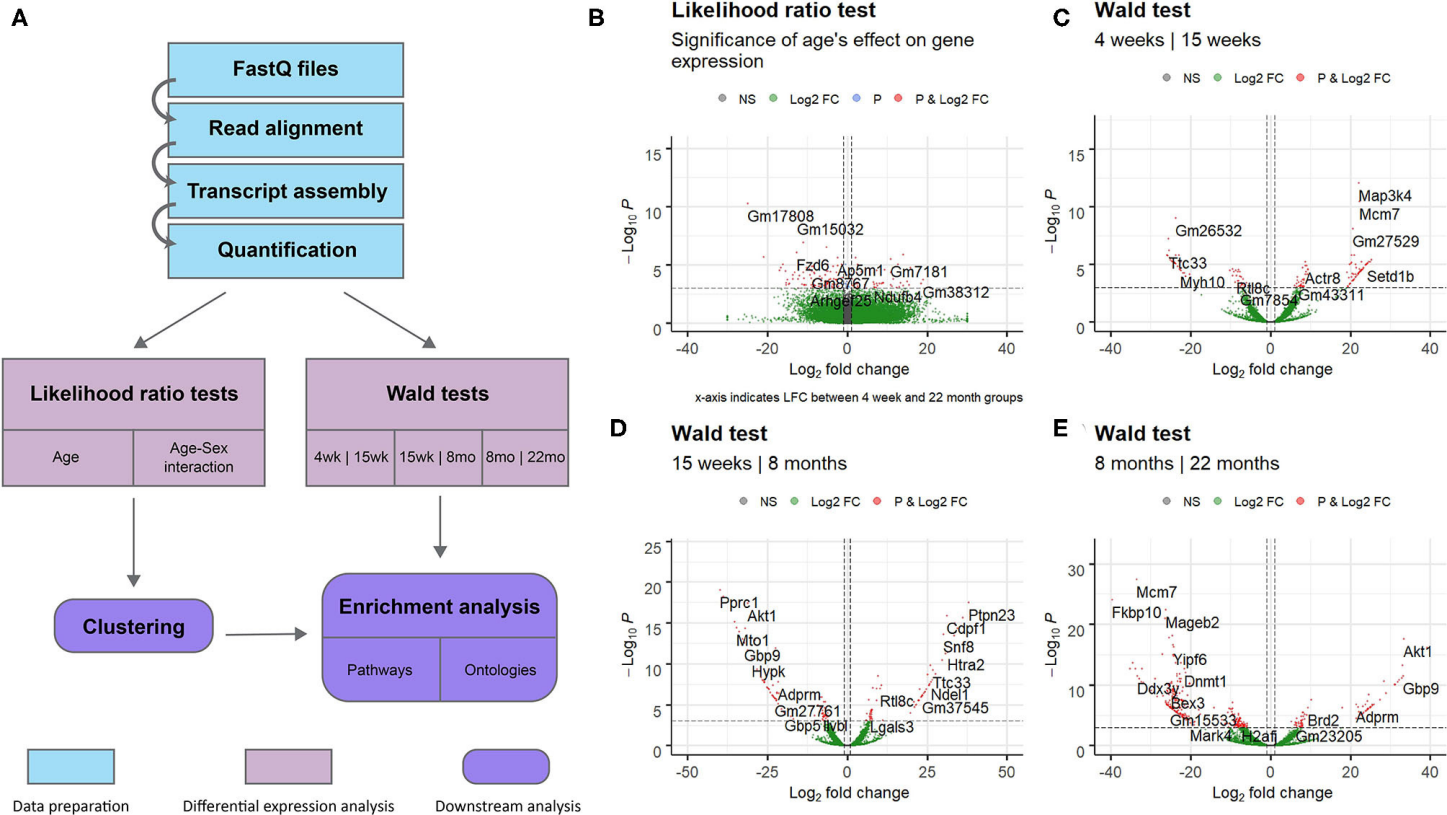
Overview of entire RNA-Seq data analysis workflow and volcano plots for dynamically expressed genes. Plots were constructed using the EnhancedVolcano (Blighe, 2019) package in R. Horizontal lines are drawn at *p* = 0.001. Vertical lines are added at log2 fold change values of 1 and −1. DESeq2's shrunken log2 fold change metric was used. Genes with significant differential expression (*p* < 0.001 and LFC >1 or LFC < −1) are colored red. **(A)** Flowchart of steps used to analyse RNA sequencing data obtained from mice of different age groups. Data preparation steps are colored in light blue, differential expression analysis steps are colored in pink, and downstream analysis steps are colored in purple. **(B)** Results of the likelihood ratio test using a reduced model with sex as the sole explanatory variable; genes identified as significant (red) vary in expression with age. **(C)** Results of the Wald test comparing gene expression in the 4 week group to expression in the 15 week group. **(D)** Results of the Wald test comparing gene expression in the 15 week group to expression in the 8 month group. **(E)** Results of the Wald test comparing gene expression in the 8 month group to expression in the 22 month group. This comparison identified the largest number of transcripts with *p* < 0.05 of the three pairwise comparisons.

This study aimed to investigate gene expression profiles of the aging mouse heart. We analyzed whole mouse heart samples at the following developmental milestones, as reviewed by Brust et al. (2015): 4 weeks of age—juvenile (too young to reproduce); 15 weeks of age—adolescent (sexually mature); 8 months of age—adult; 22 months of age—elderly (post-reproductive phase). RNA-sequencing data from wild-type mice of the four different age groups was analyzed to identify gene expression patterns associated with aging. An overview of the complete data analysis workflow, including data preparation steps, is displayed in Figure 1A. Briefly, following read quality assessment with FastQC, reads were aligned to a reference genome with STAR (Dobin et al., 2012) and assembled with StringTie (Pertea et al., 2015). 18,371 annotated transcripts with non-zero expression in at least one of the 14 mice were identified.

The full set of annotated transcripts was subjected to differential expression analysis using the likelihood ratio test (LRT) and the Wald test, producing *p*-values for each transcript, shown in Figure 1, in addition to shrunken log2 fold changes calculated by DESeq2 (Love et al., 2014). The LRT was used to assess the association of different explanatory variables with each gene's expression levels across all experimental groups. Specifically, the test was applied to identify genes whose expression was highly correlated with age and/or an interaction between age and sex. Results of the LRT used to identify genes that exhibit a significant relationship with age are shown in Figure 1B. The LRT for age identified 2,410 genes with an adjusted *p*-value < 0.05, and 147 genes with an adjusted *p*-value < 0.001 (colored red in Figure 1B). The 20 named genes with the lowest adjusted *p*-values calculated by the LRT for age are shown in Table 1. In addition, the Wald test was applied to assess the significance of transcript count differences for each gene between each pair of time points. Results of the Wald test applied to consecutive time points are displayed in Figures 1C–E. The test between 4 and 15 weeks yielded 592 genes with an adjusted *p*-value < 0.05 and 141 genes with *p* < 0.001 (Supplementary File 1). The test comparing the 15 week group to the 8 month group identified 548 genes with adjusted *p*-value < 0.05 and 154 genes with *p* < 0.001 (Supplementary File 2). The test contrasting the 8 and 22 month groups identified a larger number of DE genes, with 1,038 at adjusted *p*-value < 0.05 and 344 at *p* < 0.001 (Supplementary File 3).

**Table 1:** List of genes that exhibit dynamic expression throughout the aging process.

**Gene**	**LFC (4–15 wk)**	**LFC (15–8 mo)**	**LFC (8–22 mo)**	**Adjusted p-value**
*Fzd6*	3.79	−2.17	5.68	8.27·10^−7^
*Hs6st1*	−8.66	1.78	−8.12	1.25·10^−6^
*Slc35e3*	−7.57	−0.74	4.39	2.14·10^−6^
*Ap5m1*	3.61	−2.51	−0.96	2.52·10^−6^
*Selenok*	6.31	−0.88	10.10	2.59·10^−6^
*Mgat4a*	10.33	−6.27	−2.66	8.77·10^−6^
*Tnni1*	−3.64	0.51	7.87	8.77·10^−6^
*Ube2k*	1.66	−6.94	7.54	8.77·10^−6^
*Atmin*	−4.15	9.19	−1.08	9.09·10^−6^
*Sox6*	3.97	−7.95	−1.45	9.09·10^−6^
*Tsnax*	−3.39	−4.71	1.10	1.13·10^−5^
*Xpo7*	−5.46	2.83	0.26	1.27·10^−5^
*Hscb*	−4.59	4.00	7.07	1.78·10^−5^
*Naa10*	−1.78	0.25	14.34	1.78·10^−5^
*Ascc1*	1.10	2.22	3.95	3.01·10^−5^
*Rpl26-ps5*	0.71	2.60	2.02	3.49·10^−5^
*Rpl3l*	4.79	1.39	6.88	3.49·10^−5^
*Ddo*	−8.19	1.39	2.56	3.87·10^−5^
*Dusp19*	−4.30	1.42	−6.31	4.43·10^−5^
*Mturn*	5.23	0.62	−6.41	4.43·10^−5^

*Each table includes the top 20 genes with the lowest p-values calculated by the likelihood ratio test (LRT) for age, sorted by adjusted p-value. These p-values provide an indication of the age variable's explanatory power in the expression model for each gene. DESeq2's shrunken log2 fold change (LFC) for each pair of consecutive time points is also listed to provide information on each gene's specific expression path*.

*Fzd6, Frizzled class receptor 6; Hs6st1, Heparan Sulfate 6-O-Sulfotransferase 1; Slc35e3, Solute Carrier Family 35 Member E3; Ap5m1, Adaptor related protein complex 5 subunit mu 1; Selenok, Selenoprotein K; Mgat4a, Alpha-1,3-Manosyl-Glycoprotein 4-beta-N-acetylglucosaminyltransferase A; Tnni1, Troponin I1, slow skeletal type; Ube2k, Ubiquitin-conjugating enzyme E2 K; Atmin, ATM ineractor; Sox6, SRY-box transcription factor 6; Tsnax, Translin-associated factor X; Xpo7, Exportin-7; Hscb, HscB mitochondrial iron-sulfur cluster co-chaperone; Naa10, N-alpha-acetyltransferase 10 NatA catalytic subunit; Ascc1, Activating signal cointegrator 1 complex subunit 1; Rpl26-ps5, Ribosomal protein L26 pseudogene 5; Rpl3l, Ribosomal protein L3-like; Ddo, D-aspartate oxidase; Dusp19, dual-specificity phosphatase 19; Mturn, Maturin, neural progenitor differentiation regulator homolog*.

**Figure 2:**
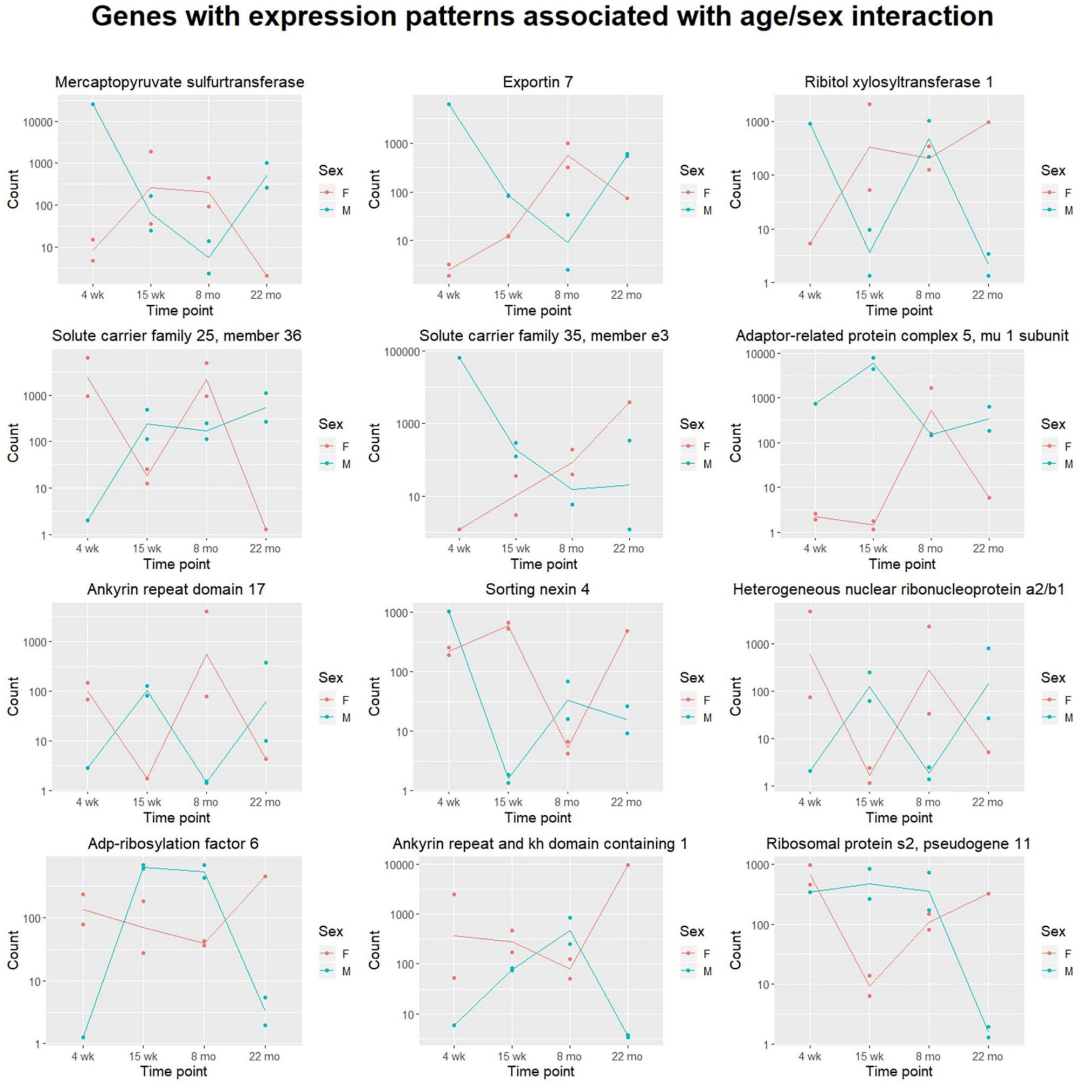
Scatter plots displaying sex-specific expression paths over time. The top 12 genes with canonical names (ranked by adjusted *p*-value) identified in the likelihood ratio test for age/sex interaction are displayed. For each gene, transcript count is displayed on the y-axis (with logarithmic scale) and age group on the x-axis. Samples from different sexes are colored differently. Expression of each of these genes is highly associated with an interaction between age and sex. Lines are added to display trajectories between mean expression levels for each sex at each time point. Lines for males (blue) and females (red) are colored differently.

We also attempted to identify genes with sex- and time-dependent expression profiles using a likelihood ratio test for age/sex interaction. The LRT for the age/sex interaction variable identified 414 genes with adjusted *p*-value < 0.05 (exact *p*-values are included in Supplementary File 5). These genes exhibit dynamic expression paths that are dependent on sex as well as age; expression not only varies between time points, but between sexes within time points. Three hundred and ninety-seven of the 414 DE genes identified in the LRT for age/sex interaction were also identified as significant in the LRT for age (adjusted *p*-value < 0.05), indicating a high level of age-dependence in their expression profiles. Expression changes over time for the 12 named genes with the lowest adjusted *p*-values calculated from the age/sex interaction LRT are displayed in Figure 2.

**Figure 3:**
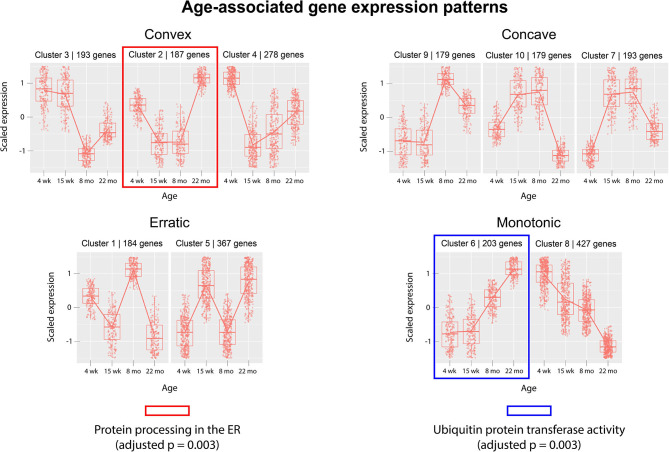
Clusters of differentially-expressed genes in the aging murine heart transcriptome. Clustering was performed using DEGreport's implementation of divisive hierarchical clustering (Pantano, 2019). Genes with adjusted *p*-value < 0.05 (calculated by the likelihood ratio test for age) were clustered according to log2 normalized read counts. Each cluster's number is provided in the title of each scaled expression graph, along with the number of genes in that cluster. Genes are plotted on the y-axis according to a scaled expression value (z-score), calculated as each gene's distance from the mean log transcript count of all genes in that cluster divided by the standard deviation of log transcript counts in that cluster. A box plot is constructed for each time point, with median expression level denoted by a horizontal line. Lines also connect the mean expression levels of consecutive time points, to display an expression path typical of that cluster. Genes that did not match the expression profiles of any of the 10 clusters were omitted. Pathway and ontology analysis was implemented on all 10 clusters, and gene sets with statistically-significant enrichment were identified in cluster 2 (red) and cluster 6 (blue). Cluster 2's genes exhibit high expression levels in both juvenile and elderly mice but are down-regulated in adolescent and adult mice, while the genes in cluster 6 exhibit a monotonic increase in expression as mice age past 15 weeks.

Next, we analyzed gene expression trajectories throughout the aging process. The 2,410 dynamically-expressed genes identified by the likelihood ratio test for age (adjusted *p*-value < 0.05) were clustered using the divisive hierarchical clustering implementation from the R package DEGreport (Pantano, 2019). The Kendall rank correlation coefficient (Kendall, 1955) was used as a distance metric. Twenty of the 2,410 genes were put into clusters with <15 members; these genes were discluded from the analysis. Each of the remaining 2,390 genes were each placed into one of 10 clusters. Figure 3 displays the expression pattern typical of each of the 10 clusters, as well as the scaled expression levels of each individual gene. Clusters were placed into one of four categories, based on their age-associated expression trajectory: clusters that exhibit a convex expression curve (3, 2, 4), those that exhibit a concave expression curve (9, 10, 7), those that exhibit erratic expression (1, 5), and those that exhibit monotonic expression changes over time (6, 8). Full clustering results, including cluster membership and p-values of individual genes, are included in Supplementary File 4. The genes in each of the 10 clusters were subjected to over-representation analysis using the R package clusterProfiler (Yu et al., 2012) to identify pathways whose genes are dynamically-regulated in consistent patterns throughout the aging process. Over-representation analysis on pathways from the Kyoto Encyclopedia of Genes and Genomes (KEGG) (Kanehisa and Goto, 2000) identified “Protein processing in the endoplasmic reticulum” (mmu04141) as significantly enriched in cluster 6 (adjusted *p*-value = 0.003). Cluster 6 shows a monotonic expression curve characterized by constant expression between 4 and 15 weeks, an increase in expression at 8 months, and a further increase at 22 months. Gene ontology (GO) enrichment analysis identified “Ubiquitin-protein transferase activity” (GO:0004842) as significantly enriched in cluster 2 (adjusted *p*-value = 0.003), a cluster with convex expression characterized by moderate expression levels at 4 weeks, low expression at 15 weeks and 8 months, and high expression at 22 months.

**Figure 4:**
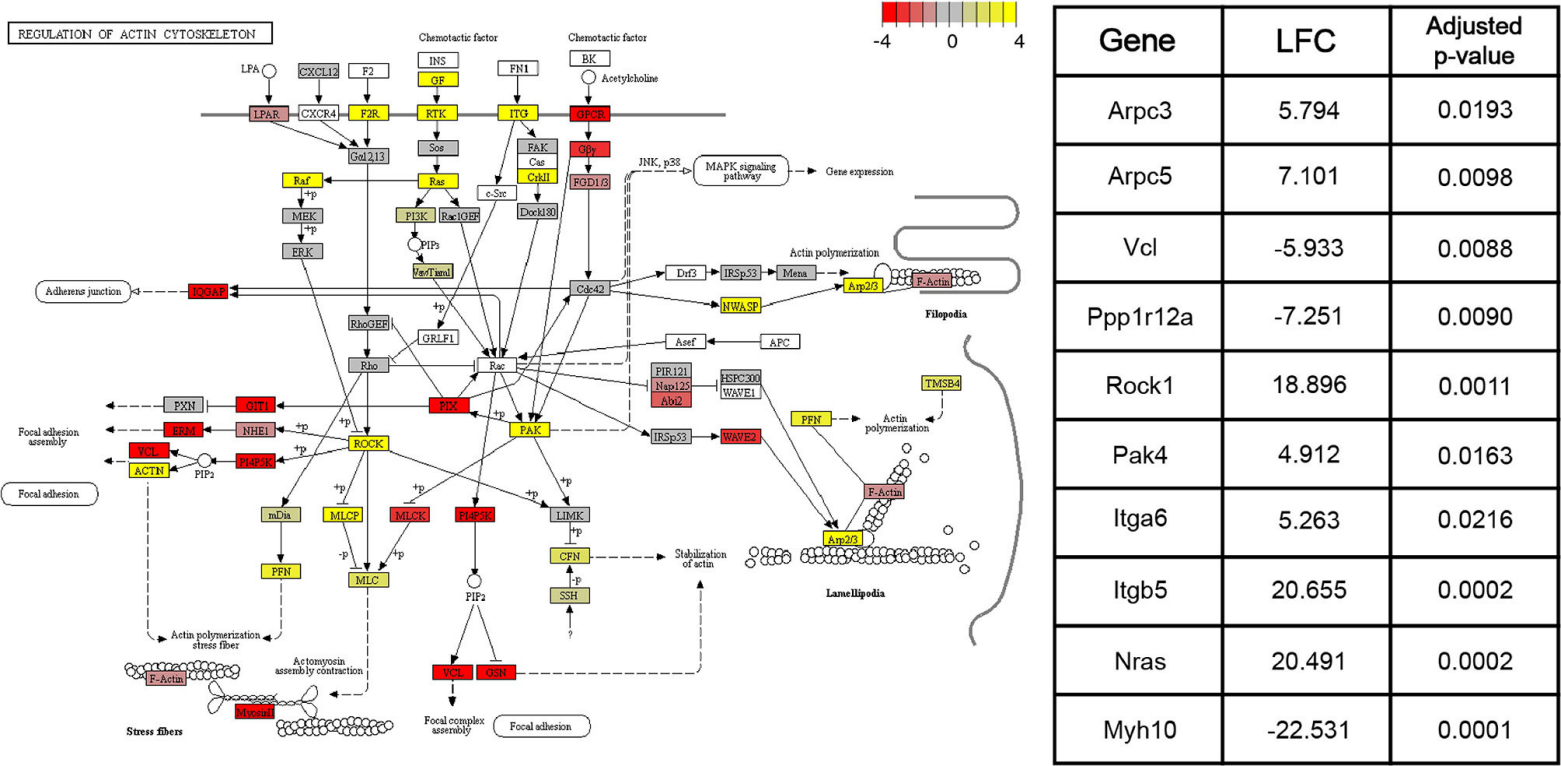
Pathway graph displaying the “Regulation of actin cytoskeleton” pathway from the KEGG (Kanehisa and Goto, 2000) database (mmu04810). This pathway was significantly activated (adjusted *p* < 0.01) between the 4 and 15 week age groups. Between the 15 week and 8 month age groups, the pathway is non-significantly inhibited (adjusted *p* = 0.09), while between the 8 month and 22 month age groups, the pathway is significantly activated (adjusted *p* < 0.05). Colors of each node correspond to the sum of DESeq2's shrunken log2 fold changes (LFCs) for genes associated with that node. Shrunken LFCs are indicated by the color scale in the top right corner. Overall, the log2 fold changes of 126 constituent genes are annotated on the pathway graph. White nodes correspond to transcripts for which read count data was not available. Some nodes represent multiple proteins with similar or redundant functions.

DE genes identified by the Wald tests between consecutive time points (adjusted *p* < 0.05) were subjected to downstream pathway analysis using the Signaling Pathway Impact Analysis (Tarca et al., 2008) (SPIA) package in R. SPIA calculates a final *p*-value of enrichment for each pathway in the KEGG (Kanehisa and Goto, 2000) database using information on over-representation, log2 fold change, and the topology of the pathway. SPIA was run using the DE genes derived from each of the three Wald tests between consecutive time points (adjusted *p*-value < 0.05), as well as their shrunken log2 fold changes. For the Wald test comparing the 4 and 15 week groups, 16 pathways with *p* < 0.05 were identified. Between the 15 week and 8 month groups, 19 such pathways were identified. Finally, between the 8 and 22 month groups, 22 significant pathways were identified. Figure 4 shows the components of the “Regulation of actin cytoskeleton” KEGG pathway (mmu04810), the pathway with the lowest adjusted *p*-value in the 4 and 15 week comparison. This pathway was identified by SPIA as significantly activated in mice of the 15 week age group compared to mice of the 4 week age group (adjusted *p* = 0.009). Ten genes in this pathway were identified as differentially-expressed (adjusted *p*-value < 0.05) between 4 and 15 weeks. Given the known role of actin remodeling in juvenile cardiac development, these data are included to demonstrate that our data and enrichment analysis techniques are able to reproduce expected results. They may also provide relevant information on age-associated expression changes that contribute to post-natal structural remodeling of the heart. Other pathways with significant enrichment between various time points including 4 and 15 weeks (“WNT signaling pathway,” Supplementary Figure 1), 15 weeks and 8 months (“VEGF signaling pathway,” Supplementary Figure 2), and 8 and 22 months (“T cell receptor signaling pathway,” Supplementary Figure 3), are included in Supplementary Figures.

Gene ontology (GO) enrichment analysis was also performed on DE genes identified by the Wald test (adjusted *p*-value < 0.05) between each pair of consecutive time points, using the R package clusterProfiler. Prior to GO enrichment analysis, DE genes were separated into those that were upregulated (log fold change >0) and downregulated (log fold change <0) between each pair of consecutive time points. Interestingly, no significantly-enriched ontologies were detected for upregulated genes between any pair of time points. Between the 4 and 15 week groups, 16 enriched GO terms (adjusted *p*-value < 0.05) were identified in the downregulated DE genes. Between the 15 week and 8 month groups, 16 enriched GO terms were identified (adjusted *p* < 0.05) in the downregulated DE genes. Between the 8 and 22 month groups, only 4 enriched terms (adjusted *p* < 0.05) were identified in the downregulated DE genes, despite the Wald test identifying a larger total number of DE genes (1,038), as well as DE genes that were downregulated in the 22 month group compared to the 4 month group (738). The exact ontologies identified by the GO enrichment analyses for the three pairwise time point comparisons are included in Supplementary Tables 4–6.

**Figure 5:**
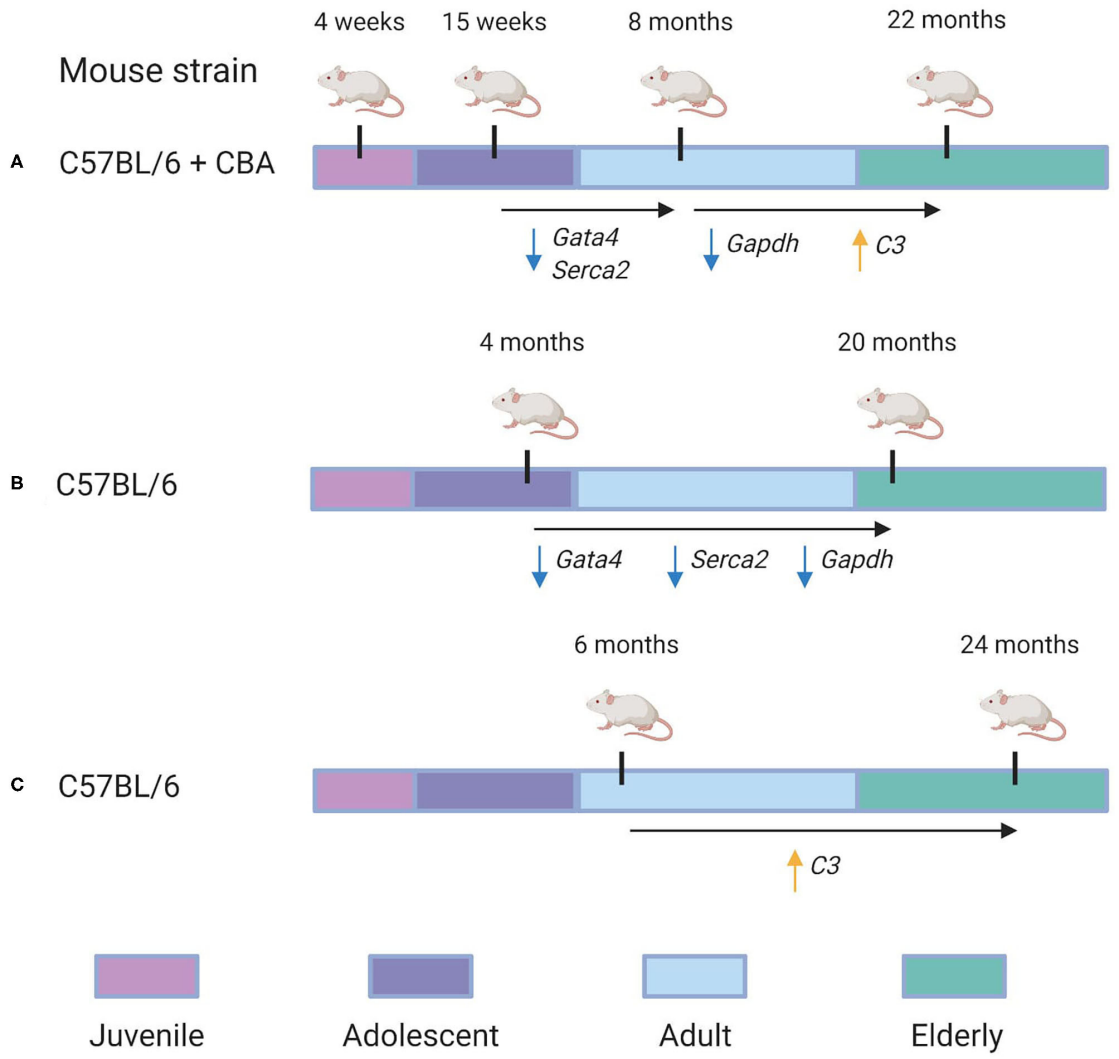
Re-evaluation of time-dependent transcriptional changes of selected transcripts. Specific genes in which conserved patterns were identified between our analysis and others are also annotated. **(A)** The experimental design used in our analysis. C57BL/6 + CBA hybrid mice of four different age groups—4 weeks, 15 weeks, 8 months, and 22 months—were analyzed to identify gene expression changes associated with aging. We identified that *Gata4* and *Serca2* were downregulated between the 15 week and 8 month groups (with LFC = −1.5 and LFC = −1.8, respectively). Furthermore, *Gapdh* was downregulated in the 22 month group compared to the 8 month group (LFC = −3.9), while *C3* was identified as upregulated in the 22 month group compared to the 8 month group (LFC = 5.1). **(B)** Overview of the experimental design used in a 2002 study by Bodyak et al. (2002). Their group compared C57BL/6 mice of 4 months age with those of 20 months age. It was identified that *Gata4, Serca2*, and *Gapdh* were all downregulated in the 20 month group compared to the 4 month group. **(C)** Overview of the experimental design used in a 2019 study by Bartling et al. (2019). They compared C57BL/6 mice of 6 months age with those of 24 months age. They identified that *C3*'s expression was significantly upregulated in the 24 month group compared to the 6 month group.

Finally, we compared our cross-sectional study with two data sets, as shown in Figure 5. Bodyak et al. (2002), for instance, compared the cardiac transcriptomes of two groups of mice: those of 4 months age and those of 20 months' age. They identified both *Gata4* and *Serca2* were identified to be downregulated by Bodyak et al. (2002) in mice of 20 months, compared to mice of 4 months age. In our data, the largest decrease in the expression of these genes was observed between 15 weeks and 8 months, with log fold change (LFC) values of −1.8 and −1.5, respectively, compared to LFC values between 0 and 1 for both genes in the 8 month/22 month comparison , implying that most of the age-associated downregulation observed in these genes occurs during adulthood rather than in elderly mice. On the other hand, the G3P dehydrogenase *Gapdh*, also identified as downregulated in mice of 20 months compared to those of 4 months (Bodyak et al., 2002), exhibited in our data its most significant downregulation in the 8-vs.-22-month comparison (LFC = −3.9), compared to the 15-week-vs.-8-month comparison (LFC = −2.4). A more recent transcriptomic study of the aging heart by Bartling et al. (2019) focused on the association of immune system components with the aging process. In accordance with their results, we identified a statistically-significant upregulation of complement factor *C3* in the 22 month group compared to the 8 month group (LFC = 5.1, adjusted *p* = 0.02). Interestingly, a downregulation in C3 expression was also observed in the 8 month group compared to the 15 week group (LFC = −3.4). Our KEGG pathway analysis of differentially-expressed genes from the 8/22 month comparison also identified a statistically significant enrichment of multiple immune response pathways, including “Chemokine signaling pathway” (mmu04082), “T cell receptor signaling pathway” (mmu04660), and “B cell receptor signaling pathway” (mmu04662) (all adjusted *p* < 0.05).

## Discussion

Aging is an intrinsic factor that makes heart tissue more susceptible to pathological stimuli and contributes to increased cardiovascular mortality and morbidity. Cardiac aging in mice recapitulates many of the molecular and physiological changes observed in aging human hearts, making rodents an effective model of human cardiac aging. In this work, we present a comprehensive statistical analysis of transcriptomic data from 14 aging murine hearts. We analyse our previously published RNA-sequencing data (Mielcarek et al., 2014) to unravel altered transcriptional signatures at physiologically-significant time points in post-natal wild type mice (on BL6C57/CBA mix genetic background). These time points were: 4 weeks of age (juvenile), 15 weeks of age (adolescent), 8 months of age (adult), and 22 months of age (elderly), as described in Mielcarek et al. (2014). An important caveat in the comparison of mice in the 8 month and 22 month groups is that natural selection might provide a biased view of age-associated expression changes if mice with certain gene expression profiles die before reaching the age of 22 months. Although the magnitude of these effects is reduced by analysing mice that are genetically-identical and raised in similar environmental conditions, the sampling bias that might result from selection should be mentioned nonetheless. In addition to our statistical analysis, we hope to demonstrate an effective re-purposing of an existing experimental data set. The wealth of publicly-available biological data provides opportunities for purely *in-silico* analyses to be conducted on published, experimentally-obtained data sets. If such data sets contain reliable information, they can be re-analyzed to produce novel findings.

This research supports ongoing efforts to characterize the transcriptional signatures of aging and development. Recent similar works have attempted to characterize differential gene expression in the fetal human heart (Pervolaraki et al., 2018), the neonatal mouse heart (Talman et al., 2018), and the elderly mouse heart (Bartling et al., 2019) but none have investigated gene expression across the entirety of the mouse lifespan. The whole-lifespan view provides greater resolution than the two group comparisons (old vs. young) traditionally used in age-related gene expression studies (Bodyak et al., 2002; Lee et al., 2002; Bartling et al., 2019). Compared to the study conducted by Bodyak et al. (2002), which compared 4 month-old mice to 20 month-old mice, our experimental design includes an additional 8 month age group between the 15 week (similar to 4 months) and 22 month (similar to 20 months) groups, allowing us to provide a more precise view of age-associated expression changes. We find that certain age-associated genes (e.g., *Gata4* and *Serca2*) identified in their experiment are downregulated between 15 weeks and 8 months in our data, while others (e.g., *Gapdh*) are downregulated between 8 months and 22 months. In line with a more recent study conducted by Bartling et al. (2019), we identified an upregulation of immune system components and pathways as associated with the transition from 8 to 22 months.

The gene with the lowest *p*-value (LFC = 22.1, adjusted *p* = 8.6 · 10^−13^) in the comparison between adolescents (15 weeks) and juveniles (4 weeks) was Mitogen-activated protein kinase kinase kinase 4 (*Map3k4*), a MAP-kinase that activates the transcription factors c-Jun N-terminal kinase (JNK) and P38 in response to stress stimuli (Bettinger and Amberg, 2007). *Map3k4*'s expression was significantly upregulated in the 15 week mice, perhaps indicating a response to oxidative stress resulting from the metabolic adaptations made to accommodate the increased energetic demands of the growing heart. The switch to oxidative metabolism in the postnatal heart appears to be necessary for healthy development, as the inactivation of factors regulating mitochondrial metabolic activity has been shown to induce pathological remodeling in the developing heart (Papanicolaou et al., 2012). The changes in metabolic profile associated with normal heart growth may therefore be sufficient to explain *Map3k4*'s upregulation between juvenile and adolescent stages. Interestingly, other work has indicated that pressure overload, a key driver of pathological hypertrophy, increases MAP3K4's kinase activity (Mizote et al., 2010). Mizote et al. (2010) also showed that H_2_O_2_ increases MAP3K4 protein levels in a dose-dependent manner. Oxidative stress has been identified as a key feature of pathological hypertrophy (Maulik and Kumar, 2012), suggesting that an upregulation of *Map3k4*'s activity may therefore also be observed under those circumstances. Although the phenotypic features of physiological and pathological forms of cardiac hypertrophy are distinct (Shimizu and Minamino, 2016), these results indicate that a transcriptional upregulation of *Map3k4* may underscore both processes. Targeted investigations of the kinase's role in normal cardiac development may provide mechanistic insights into similarities between healthy and pathological forms of cardiac remodeling.

Another gene significantly upregulated in the 15 week group compared to the 4 week group was *Akt1* (LFC = 19.8, adjusted *p* = 9.3·10^−6^), a multi-functional kinase whose expression in the cardiovascular system is well-documented (O'Neill and Abel, 2005; Chaanine and Hajjar, 2011). In the postnatal heart, *Akt1* appears to mediate physiological hypertrophy. For instance, in dominant-negative transgenic mouse models, the kinase has been demonstrated to be necessary for healthy insulin-dependent heart growth (Shiojima et al., 2002). Interestingly, this gene also produced a highly significant p-value in the comparison between adult mice (8 months) and elderly mice (22 months), being significantly upregulated in the 22 month group compared to the 8 month group (LFC = 33.4, adjusted *p* = 2.3·10^−18^). Other evidence in transgenic mouse models has indicated that *Akt1* overexpression is associated with a broad range of pathological hypertrophic phenotypes in the hearts of adult mice, depending on the duration of overexpression (Nagoshi et al., 2005; Shiojima et al., 2005). Haploinsufficiency of *Akt1* has also been shown to increase mouse lifespan (Nojima et al., 2013). These results again suggest common molecular mechanisms between physiological and pathological forms of cardiac hypertrophy. Here, we provide evidence that in wild-type mice, an increase in *Akt1* expression is associated with progression both from adulthood into old age and from juvenility into adolescence. Our findings would be well-supplemented with a more detailed view of the morphological changes that accompany the age-associated expression changes of *Akt1*.

The likelihood ratio test (LRT) was implemented to identify genes exhibiting significant expression variation across the four age groups, rather than in any single pairwise comparison. Interestingly, this method identified a much larger number of age-associated genes than were discovered in a recent meta-analysis, which may reflect the contribution of strain-specific effects (Palmer et al., 2019). Although the LRT lacks the resolution of pairwise comparisons between age groups, it served as an effective filter for our downstream clustering analysis, intended to cluster genes based on their age-dependent expression patterns. We implemented divisive hierarchical clustering using a rank correlation coefficient as a dissimilarity metric, aiming to group genes by relative expression trajectories rather than absolute transcript count profiles. We hypothesized that a non-parametric distance metric would be more robust to differences in the baseline expression levels of biologically-linked components, while still extracting information about their concurrent expression changes between time points. The clustering identified a variety of gene expression patterns associated with age, including linear and non-linear trajectories. These gene expression patterns were grouped into four categories by visual inspection: monotonic (linear), convex, concave, and erratic. The convex and concave trajectories are of particular interest, as they suggest that certain juvenile gene expression programs may be recapitulated in the hearts of older mice. The re-activation of juvenile gene expression programs in old age has been observed in transcriptomic studies of neural tissue (Somel et al., 2010; Colantuoni et al., 2011) and in models of cardiac hypertrophy (Dirkx et al., 2013). However, to date, this relationship has not been demonstrated in any published transcriptional analyses of aging cardiac tissue. Here, we provide evidence that similar gene expression patterns characterize the cardiac aging process in mice.

Gene clusters were analyzed for enriched annotations to reveal biological processes associated with different expression trajectories. Over-representation analysis of the 10 gene clusters using KEGG pathways identified “Protein processing in the endoplasmic reticulum” (mmu04141) as an over-represented pathway (adjusted *p* = 0.003) in cluster 6, a monotonic cluster. Genes in this cluster tend to exhibit a steady increase in expression throughout the lifespan of the mouse and are uniquely expressed at high levels in elderly mice. Recent work has detected increased levels of protein aggregates in cardiac tissue from both elderly and hypertensive mice, as well as identifying significant overlap in the constituent proteins of the isolated aggregates (Ayyadevara et al., 2016). Our results suggest that a transcriptional upregulation of ER-associated components accompanies—and may act a response to—the age-associated accumulation of protein aggregates in cardiac tissue.

Over-representation analysis of the same 10 clusters using gene ontology annotations also identified “Ubiquitin protein transferase activity” (GO:0004842) as significantly enriched in cluster 2 (adjusted *p* = 0.003). Similar to cluster 6, genes in cluster 2 are characterized by high expression levels in the 22 month age group, yet unlike cluster 6, cluster 2's genes are also expressed at high levels in the 4 week group. The gene with the lowest *p*-value (adjusted *p* = 0.0008) associated with this GO term in cluster 2 was Tumor necrosis factor receptor associated factor 6 (*Traf6*). *Traf6*'s mean expression was highest in the 22-month group (4,753 transcripts) and lowest in the 15-week group (11 transcripts). A strong association between increased Traf6 expression and pathological hypertrophy in both mice and humans was established in a 2016 experiment (Ji et al., 2016) that noted a >2-fold increase in *Traf6*'s expression in human dilated cardiomyopathy hearts compared to normal controls. Our data indicate that increased *Traf6* expression is associated with the natural aging process in the wild-type murine heart. Our clustering results also suggest more generally that ubiquitin-transferase activity may be associated with age-related changes in the heart. Interestingly, aggregation of polyubiquitinated proteins has been shown to trigger autophagy in cardiomyocytes (Tannous et al., 2008), and cardiomyocyte autophagy has been separately implicated in the pathogenesis of heart failure. A 2007 experiment demonstrated that downregulation of autophagic activity reduces the pathological effects of hemodynamic stress, while upregulation has the opposite effect (Zhu et al., 2007). Although age-associated polyubiquitinated aggregates may accumulate due to defects in cellular proteolytic systems, these results also indicate that a transcriptional upregulation of genes involved in ubiquitin transfer is also associated with aging. This response may contribute to the formation of poly-Ub aggregates and the induction of cardiomyocyte autophagy. Thus, our results suggest that a transcriptional response to protein accumulation in cardiac tissue constitutes a key feature of the aging process in wild-type mice.

The likelihood ratio test was also implemented to identify genes with expression patterns dependent on age-sex interaction. These genes exhibit expression patterns that vary over time and between sexes: male-associated at some ages, and female-associated at others. Although other work has investigated sex-related gene expression differences in cardiac tissue (Isensee et al., 2007), no published literature has investigated gene expression differences associated with interactions between age and sex. Although our experiment was limited to sample sizes of one to two mice per age/sex combination, hypothesis tests identified a number of potentially-interesting genes for further investigation. Shown in Figure 4, Ribitol xylosyltransferase 1 (*Rxylt1*) was identified as differentially expressed (adjusted *p* = 0.002) by the LRT for age-sex interaction. For males, *Rxylt1* exhibited high expression at 4 weeks and 8 months but low expression in the 15 week and 22 month group. Females, conversely, exhibited low expression at 4 weeks and high expression from 15 weeks onwards. RXYLT1's glycosylation target, α-dystroglycan, is known to be associated with certain muscular dystrophies (Endo, 2014). Defects in multiple dystroglycan glycosyltransferases have been linked to dystrophic phenotypes (Muntoni et al., 2008), including LARGE1, another xylotransferase that acts on α-dystroglycan (Brockington et al., 2005). In aging mouse hearts, dystroglycan ablation has been demonstrated to produce activity-induced cardiomyopathy by disrupting extracellular matrix interactions, beginning at around 7 months of age (Michele et al., 2009). The authors posited that the dystroglycan glycosylation deficiencies observed in human muscular dystrophy patients may produce similar effects by interfering with matrix receptor function. In addition to age-dependence, our results indicate some level of sex-dependence in the expression of *Rxylt1*. These results may be relevant to investigations regarding the sex-related differences observed in dystroglycanopathies (Fanin et al., 2014). The LRT for age-sex interaction also identified two members of the six-transmembrane-helix mitochondrial SLC25 (solute carrier family 25 and 35) family as significant. The latter members are involved in transport of solutes across the inner mitochondrial membrane, namely Solute carrier family 25 member 36 (*Slc25a36*) and Solute carrier family 35 member e3 (*Slc35e3*) (Gutiérrez-Aguilar and Baines, 2013) and are deregulated in an age- and sex- dependent manner (adjusted *p* = 0.002 and adjusted *p* = 0.003, respectively). Interestingly, *Slc35e3* was found to be up-regulated during cardiac hypertrophy (Meng et al., 2018), while *Slc25a36* has been previously identified to be involved in the metabolism of nucleic acids in cardiac cells (Ogunbona and Claypool, 2019). Finally, *Snx4* (Sortin nexin 4) transcripts also exhibited age- and sex-associated expression (adjusted *p* = 0.004), with the largest difference between sexes observed in the 15 week group. The family of sorting nexins consists of a diverse group of cytoplasmic and membrane bound proteins that play a role in protein trafficking (Yang et al., 2019). In particular, SNX4 has been shown to interact with β-site amyloid precursor protein cleaving enzyme 1 (BACE1) and prevents its lysosomal degradation, leading to increased production of β-amyloids (Kim et al., 2017). Interestingly, plasma BACE1 levels have been found to be significantly different in women and men (Vergallo et al., 2019).

Overall, this analysis aims to provide a high-level overview of the transcriptomic features of aging and development. We identify genes, pathways, and gene ontologies that are associated with various developmental transitions in the murine lifespan. Two genes of particular interest are *Map3k4* and *Akt1*, both of which were detected as key markers of the transition from the 4 to the 15 week groups and both of which have been suggested as markers of pathological hypertrophy. We also identify multiple genes with expression patterns that are associated with an interaction between age and sex, which may provide useful information on how biological sex influences the effects of aging. Finally, we apply an unsupervised machine learning approach to extract common expression trajectories from the expression profiles of 2,000+ age-associated genes. Our results reveal four categories of expression trajectory within our set of age-associated genes: monotonic, convex, concave, and erratic. These findings support the notion that aging is characterized both by the re-activation of gene expression programs from earlier life and the induction of novel expression programs. Further investigation into the molecular features of long-term cardiac development may reveal other interesting findings.

## Materials and Methods

### Data Set

Read data from a 2014 study by Mielcarek et al. (2014) were obtained from the Gene Expression Omnibus database (accession code GEO58996) and subjected to statistical analysis. All samples analyzed were obtained from wild-type (WT) mice of B6CBAF1 genetic background. Sequencing was performed by Expression Analysis on an Illumina Hi-seq 2000. Paired-end sequencing was obtained, 4-plexed across lanes for a minimum of 38 million 50 mer paired reads per sample. The full data set comprised 14 mice from four different age groups: three mice at 4 weeks old, four mice at 15 weeks old, four mice at 8 months old, and three mice at 22 months old. One male and two females comprised the 4 week group, two males and one female the 22 month group, and two males and two females the 15 week and 8 month groups. In total, the set of 14 mice consisted of seven males and seven females. Two technical replicates were obtained for each mouse, producing a total of 28 samples for analysis.

### Data Preparation

Raw fastq reads were aligned to the comprehensive, indexed M22 Mus musculus genome (GrCm38.p6), obtained from Gencode. STAR (Dobin et al., 2012) was used to align raw reads to the genome. Then, StringTie (Pertea et al., 2015) was used to assemble transcripts from the mapped reads. Using the Python 2.7 script released by the creators of StringTie, count data were generated across all samples for all assembled transcripts. These data were imported into R, where differential expression (DE) analysis was performed.

Count data were filtered by removing transcripts that were not detected in any of the 28 samples. Unannotated transcripts were also filtered from the count data. Exploratory hierarchical clustering was performed to verify clustering of technical replicates, and one outlier was identified: a technical replicate of the male mouse in the 4 week group. This sample was removed from the data set, and the technical replicates for all other mice were averaged to produce a reduced data set of annotated transcript counts for 14 mice.

### Differential Expression Analysis

The R package DESeq2 was used to analyse differential expression. According to the process described by Love et al. (2014), DESeq2 models count data using the following Negative Binomial distribution:

$$K_{ij}\sim \text{NB}\left(\mu_{ij}=s_{ij}q_{ij},\alpha_{i}\right)$$

where *K*_*ij*_ is the read count for gene *i* in sample *j*. Parameters μ and α are estimated using a generalized linear model (GLM) with a logarithmic link function:

$$\log q_{ij}=\underset{r}{\sum }x_{jr}\beta_{ir},$$

where *r* indexes columns in the design matrix, which correspond to different conditions in our experiment (values of age/sex across the 14 mice).

The GLM is fit to un-normalized read counts, and normalization constants (*s*_*ij*_) are estimated using a median-of-ratios method to derive the final μ estimate for each distribution. The α parameter, related to a gene's expression variance, is estimated by fitting a curve across all genes, modeling the relationship between an initial α estimate and mean read count for each gene. The final α estimate for each gene is calculated by shrinking the gene's initial estimate toward the curve—i.e., by using information across all genes of similar expression strength to derive a more reliable estimate for expression variance. DESeq2 also performs shrinkage on β estimates, using a zero-centered normal distribution as a prior to shrink values toward zero. This results in adjusted (shrunken) log2 fold changes, which are less sensitive to changes in expression levels for genes with low transcript counts. To account for the large number of tests being performed, DESeq2 uses the Benjamini-Hochberg procedure (Benjamini and Hochberg, 1995) with a false discovery rate of 0.1 to calculate an adjusted *p*-value for each statistical test.

#### Pairwise Comparisons

To analyse expression differences between age groups, DESeq2's Wald test was implemented. The Wald test calculates a *p*-value of observing a condition's association with a gene's expression, under the null hypothesis that the condition's corresponding GLM coefficient, β_*ir*_, divided by its standard error, is distributed under a standard normal distribution. To contrast the effects of two levels of a condition, DESeq2 estimates a *contrast* β *coefficient*, $\beta_{i}^{c}$. $\beta_{i}^{c}$ is calculated as the difference between β_*i*_ estimates for two conditions, and a two-tailed *p*-value is again calculated under the null hypothesis:

$$\frac{\beta_{i}^{c}}{\text{SE}\left(\beta_{i}^{c}\right)}\sim N\left(\mu =0,\sigma^{2}=1\right)$$

where SE($\beta_{i}^{c}$) is the coefficient's standard error. DESeq2 multiplies the tail integrals of the normal distribution by 2 to achieve a two-tailed test. Genes with adjusted *p* < 0.05 (after this multiplication) were identified as significant in each pairwise comparison, corresponding to an α level of 0.05.

#### Model Comparisons

To identify genes whose expression was significantly associated with different age-related variables, the likelihood ratio test (LRT) statistic was computed using DESeq2 (Love et al., 2014):

$$t(k)=\frac{sup_{\theta \in \Theta }\;L(\theta ;k)}{sup_{\theta \in \Theta_{0}}\;L(\theta ;k)},$$

where *L* denotes a likelihood function, Θ is the set of parameters in the full model, and Θ_0_ is the set of parameters in a reduced model for a random vector *k*—in our experiment a vector of transcript counts for a single gene. We used a full model containing age group, sex, and interaction coefficients and generated two different reduced models by removing the coefficients associated with different explanatory variables in the full model. One reduced model was generated by removing all age-related terms from the full model (LRT for age) while the other reduced model was generated by removing only the age/sex interaction term (LRT for age/sex interaction). By noting that $-2\log t\overset{d}{\underset{}{\to }}\chi_{f}^{2}$, where *f* is the difference in the number of independent parameters between the full and reduced models, both LRTs calculate a one-tailed *p*-value for each gene. For the variable removed from each model, this *p*-value expresses the importance of that variable in explaining the expression level of each gene. Using this method, genes with adjusted *p*-values < 0.05 were identified as significant, corresponding to an α level of 0.05.

#### Visualization

To visualize the differentially-expressed (DE) genes identified using the methods described above, the R packages EnhancedVolcano (Blighe, 2019) and gplots (Warnes et al., 2019) were used. EnhancedVolcano was used to display each gene's shrunken log2 fold change (LFC) against its adjusted *p*-value.

### Clustering

The R package DEGreport (Pantano, 2019) was used to perform hierarchical clustering on differentially-expressed genes (adjusted *p* < 0.05) identified by the LRT for age. DEGreport calculates the similarity between the expression profiles of each pair of DE genes by computing a Kendall correlation coefficient, τ, between their mean expression values in different age groups. For any two genes, each with an associated ranking of age groups based on mean expression level within each age group:

$$\begin{array}{l} \tau =\\ \;\frac{\text{(number of concordant pairs)}-\text{(number of discordant pairs)}}{\left(\begin{array}{c} n\\ 2 \end{array}\right)}, \end{array}$$

where a concordant pair corresponds to two age groups with the same relative expression relationship for both genes—either higher or lower expression in the same age group for both genes. A gene-gene distance matrix is constructed from the pairwise Kendall coefficients, from which gene clusters are created by divisive analysis (DIANA) of hierarchical clustering (Kaufman and Rousseeuw, 1990). This method generates a series of hierarchical similarity relationships as different data points are sequentially split to form new clusters, creating a dendrogram. The degPatterns function cuts the dendrogram at height equal to divisive coefficient of the clustering, a measure of clustering structure in the data set. In effect, this generates a lower number of clusters for data with tighter clustering structure. This method was applied to create clusters of genes with similar expression paths over time. These clusters were subjected to enrichment analysis.

### Enrichment Analysis

#### Pathway Analysis

The R package Signaling Pathway Impact Analysis (Tarca et al., 2008) (SPIA) was used to identify pathways that were significantly up- or down-regulated between each pair of time points. Enriched pathways were identified using DE genes highlighted by the Wald test (adjusted *p* < 0.05). SPIA uses a combination of over-representation and topology-based analyses to estimate two *p*-values for each pathway. SPIA uses the hypergeometric test to calculate a *p*-value for enrichment and calculates a separate perturbation *p*-value based on the log fold changes of pathway components and the network's topology. These *p*-values are combined using Fisher's method. SPIA uses pathway annotations from the KEGG (Kanehisa and Goto, 2000) database. Pathway data were visualized using the R package Pathview (Luo et al., 2013).

#### Ontology Analysis

The R package clusterProfiler (Yu et al., 2012) was used to perform gene ontology (GO) enrichment analysis on DE genes identified by the three pairwise age group comparisons using a hypergeometric test. Genes that were upregulated (log fold change >0) and downregulated (log fold change <0) between each pair of time points were analyzed separately for enrichment. Information on biological process (BP), molecular function (MF), and cellular compartment (CC) subontologies was collected. Enriched ontologies were defined as those with an adjusted *p* < 0.05 after Benjamini-Hochberg correction with false discovery rate 0.1. clusterProfiler was also used for enrichment analysis (pathway and gene ontology) of the gene clusters created using DEGreport (Pantano, 2019).

## Data Availability Statement

All datasets generated for this study are included in the article/Supplementary Material.

## Author Contributions

MG, AM, MI, and MM designed the study. MG and AM performed the analysis. MG, AM, DH, MI, and MM drafted the manuscript. All authors contributed to the article and approved the submitted version.

### Conflict of Interest

The authors declare that the research was conducted in the absence of any commercial or financial relationships that could be construed as a potential conflict of interest.
