# Supplementary material for: Cross-Sectional Transcriptional Analysis of the Aging Murine Heart
Source: Front Mol Biosci. 2020 Sep 25;7:565530. doi: 10.3389/fmolb.2020.565530 (PMC7545256; doi:10.3389/fmolb.2020.565530)
Supplement: Supplementary file 1 [file Data_Sheet_1.PDF]

Supplementary Materials

SUPPLEMENTARY DATA

Supplementary file 1. Full list of genes identified as significant (adjusted $p < 0.05$) by the Wald test between the 4 weeks and 15 weeks age groups. 592 genes were identified as significant by the comparison between these age groups. Shrunken log2 fold changes and adjusted p-values are included for each gene.

Supplementary file 2. Full list of genes identified as significant (adjusted $p < 0.05$) by the Wald test between the 15 weeks and 8 months age groups. 548 genes were identified as significant by the comparison between these age groups. Shrunken log2 fold changes and adjusted p-values are included for each gene.

Supplementary file 3. Full list of genes identified as significant (adjusted $p < 0.05$) by the Wald test between the 8 months and 22 months age groups. 1038 genes were identified as significant by the comparison between these age groups. Shrunken log2 fold changes and adjusted p-values are included for each gene.

Supplementary file 4. Full list of genes identified as significant (adjusted $p < 0.05$) by the likelihood ratio test (LRT) for age. 2410 genes were identified as significant by the LRT for age, of which 2390 were clustered. Cluster membership and adjusted p-values are included for each gene.

Supplementary file 5. Full list of genes identified as significant (adjusted $p < 0.05$) by the likelihood ratio test (LRT) for age-sex interaction. 414 genes were identified as significant by the LRT for age. Adjusted p-values are included for each gene.

SUPPLEMENTARY FIGURES

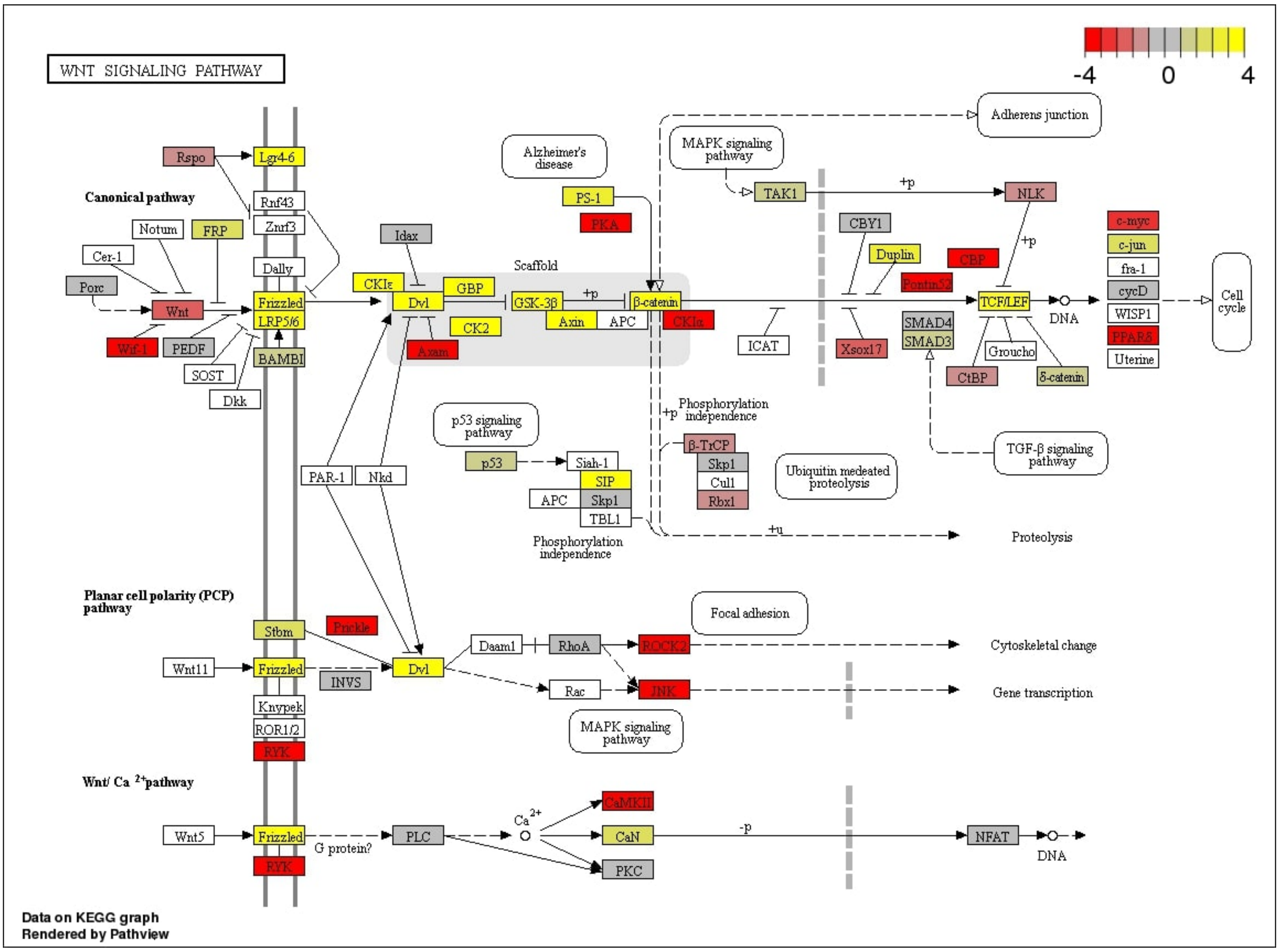

Figure S1. Pathway graph displaying the 'Wnt signaling pathway' from the KEGG database (Kanehisa and Goto, 2000). This pathway was significantly up-regulated (adjusted $p < 0.05$) between the 4 week and 15 week age groups. The Wnt pathway was non-significantly inhibited between the 15 week and 8 month time points ($p = 0.14$) and non-significantly activated between the 8 month and 22 month age groups ($p = 0.69$). Colours of each node correspond to the sum of DESeq2's shrunken log2 fold changes (LFCs) for genes associated with that node. Shrunken LFCs are indicated by the colour scale in the top right corner. White nodes correspond to transcripts for which read count data was not available. Some nodes represent multiple proteins with similar or redundant functions.

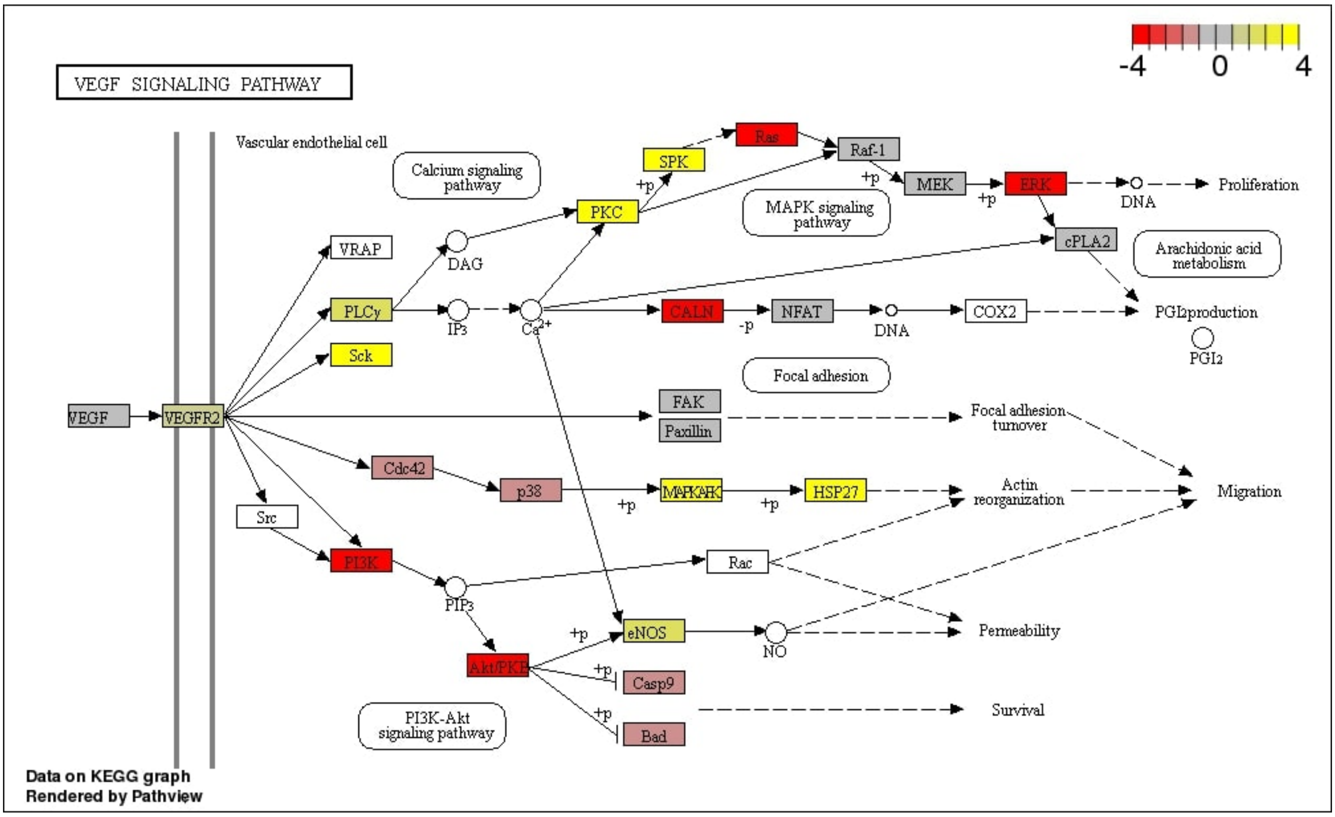

Figure S2. Pathway graph displaying the 'VEGF signaling pathway' from the KEGG database (Kanehisa and Goto, 2000). This pathway was significantly up-regulated (adjusted $p < 0.05$) between the 15 week and 8 month age groups. The pathway was non-significantly activated between the 4 week and 15 week time points ($p = 0.06$) and significantly activated between the 8 month and 22 month time points ($p < 0.01$). Colours of each node correspond to the sum of DESeq2's shrunken log2 fold changes (LFCs) for genes associated with that node. Shrunken LFCs are indicated by the colour scale in the top right corner. White nodes correspond to transcripts for which read count data was not available. Some nodes represent multiple proteins with similar or redundant functions.

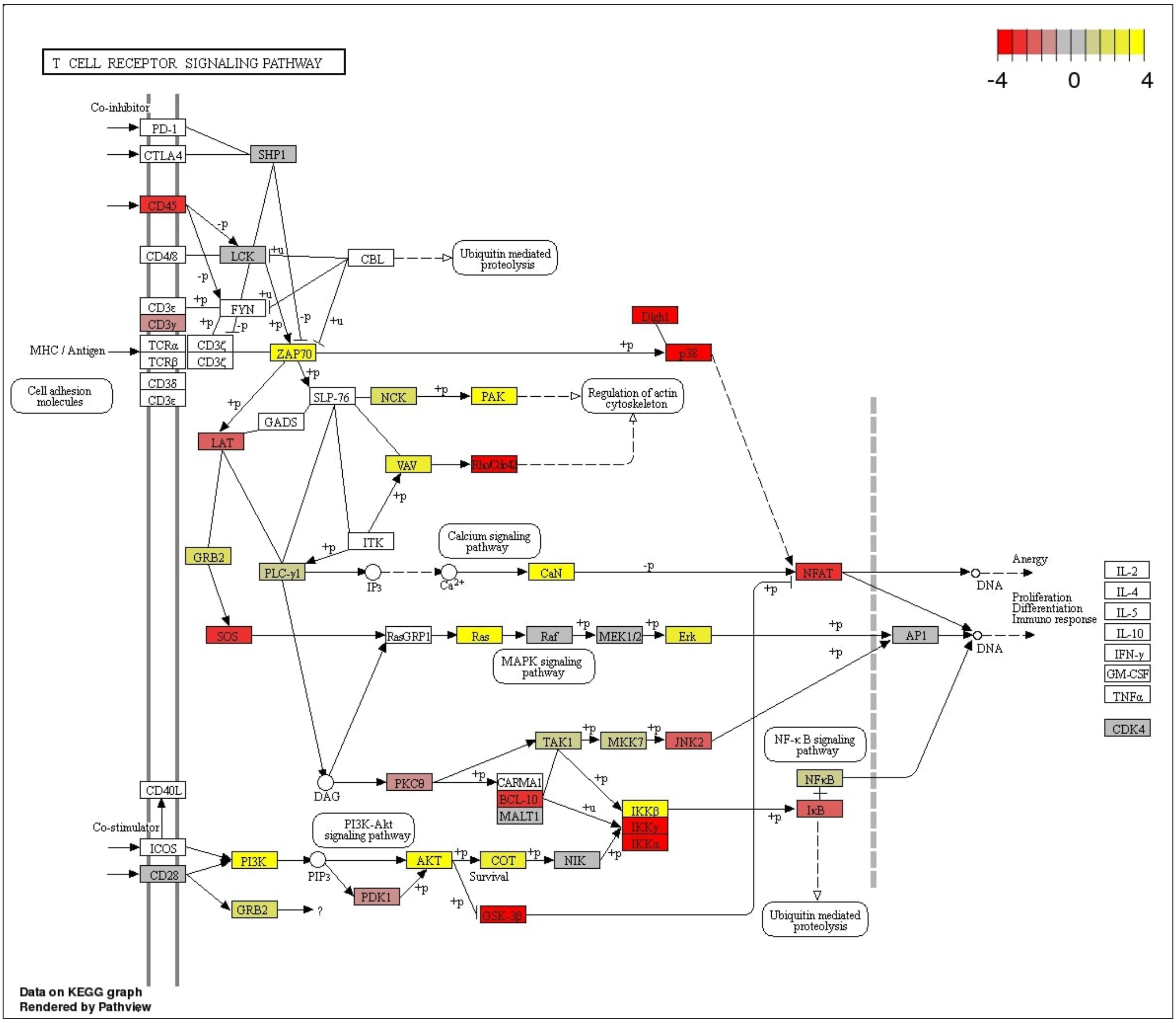

Figure S3. Pathway graph displaying the 'T cell receptor signaling pathway' from the KEGG database (Kanehisa and Goto, 2000). This pathway was significantly up-regulated (adjusted $p < 0.01$) between the 8 month and 22 month age groups. The pathway was also significantly up-regulated between the 4 week and 15 week age groups ($p < 0.05$), and was significantly down-regulated between the 15 week and 8 month age groups ($p < 0.05$). Colours of each node correspond to the sum of DESeq2's shrunken log2 fold changes (LFCs) for genes associated with that node. Shrunken LFCs are indicated by the colour scale in the top right corner. White nodes correspond to transcripts for which read count data was not available. Some nodes represent multiple proteins with similar or redundant functions.

SUPPLEMENTARY TABLES

Gene	log2 fold change	Adjusted p-value	Gene	log2 fold change	Adjusted p-value
Map3k4	22.14	$8.57 \cdot 10^{-13}$	*Cerk*	-25.58	$5.94 \cdot 10^{-8}$
Mcm7	22.21	$3.51 \cdot 10^{-11}$	*Ttc33*	-25.38	$6.26 \cdot 10^{-7}$
Mkrn2	20.3	$1.44 \cdot 10^{-6}$	*Plec*	-22.93	$1.44 \cdot 10^{-6}$
Hypk	25.19	$4.00 \cdot 10^{-6}$	*Sult1a1*	-23.71	$1.44 \cdot 10^{-6}$
Nr2c2ap	24.61	$6.05 \cdot 10^{-6}$	*Timm10b*	-25.91	$1.44 \cdot 10^{-6}$
Pgam5	22.69	$6.05 \cdot 10^{-6}$	*Srpk2*	-25.84	$1.46 \cdot 10^{-6}$
Fkbp10	20.31	$6.83 \cdot 10^{-6}$	*Mir27b*	-25.69	$1.69 \cdot 10^{-6}$
Setd1b	24.14	$8.47 \cdot 10^{-6}$	*Pcgf5*	-25.34	$2.60 \cdot 10^{-6}$
Sestd1	20.13	$8.92 \cdot 10^{-6}$	*Pam*	-25.06	$3.64 \cdot 10^{-6}$
Akt1	19.78	$9.32 \cdot 10^{-6}$	*Ndel1*	-24.75	$4.97 \cdot 10^{-6}$
Cracr2b	23.93	$9.32 \cdot 10^{-6}$	*Ghdc*	-24.31	$6.05 \cdot 10^{-6}$
Mto1	23.92	$9.32 \cdot 10^{-6}$	*Mark4*	-24.42	$6.05 \cdot 10^{-6}$
Zmat5	21.26	$9.67 \cdot 10^{-6}$	*Med16*	-24.43	$6.05 \cdot 10^{-6}$
Pprc1	23.85	$9.71 \cdot 10^{-6}$	*Med19*	-24.33	$6.05 \cdot 10^{-6}$
Tut1	23.77	$1.06 \cdot 10^{-5}$	*Kcnh2*	-24.14	$6.83 \cdot 10^{-6}$
Actr8	8.72	$1.22 \cdot 10^{-5}$	*Piezo1*	-24.17	$6.83 \cdot 10^{-6}$
Dmac2l	21.39	$1.78 \cdot 10^{-5}$	*Bak1*	-24.05	$7.45 \cdot 10^{-6}$
Cltc	23.24	$1.86 \cdot 10^{-5}$	*Aqr*	-23.85	$8.81 \cdot 10^{-6}$
Cybc1	23.02	$2.33 \cdot 10^{-5}$	*Exoc5*	-23.84	$8.81 \cdot 10^{-6}$
Hba-a2	22.22	$2.33 \cdot 10^{-5}$	*Tmbim1*	-9.48	$2.53 \cdot 10^{-5}$

Table S1. List of genes that exhibit significant up-regulation (left) or down-regulation (right) in early age. Each table includes the top 20 up- or down-regulated genes with the lowest p-values calculated by the Wald test between the 4 and 15 week age groups, sorted by adjusted p-value. The Benjamini-Hochberg multiple testing correction was used to adjust p-values. The p-values provide an indication of the magnitude of expression differences for each gene between the two time points. DESeq2's shrunken log2 fold change (LFC) for each gene is also listed.

Upregulated transcripts: *Map3k4*, mitogen-activated protein kinase kinase kinase 4; *Mcm7*, minichromosome maintenance complex component 7; *Mkrn2*, makorin, ring finger protein, 2; *Hypk*, huntingtin interacting protein K; *Nr2c2ap*, nuclear receptor 2C2-associated protein; *Pgam5*, phosphoglycerate mutase family member 5; *Fkbp10*, FK506 binding protein 10; *Setd1b*, SET domain containing 1B; *Sestd1*, SEC14 and spectrin domains 1; *Akt1*, thymoma viral proto-oncogene 1; *Cracr2b*, calcium release activated channel regulator 2B; *Mto1*, mitochondrial tRNA translation optimization 1; *Zmat5*, zinc finger, matrin type 5; *Pprc1*, peroxisome proliferative activated receptor, gamma, coactivator-related 1; *Tut1*, terminal uridylyl transferase 1, U6 snRNA-specific; *Actr8*, ARP8 actin-related protein 8; *Dmac2l*, distal membrane arm assembly complex 2 like; *Cltc*, clathrin, heavy polypeptide (Hc); *Cybc1*, cytochrome b 245 chaperone 1; *Hba-a2*, hemoglobin alpha, adult chain 2.

Downregulated transcripts: *Cerk*, ceramide kinase; *Ttc33*, tetratricopeptide repeat domain 33; *Plec*, plectin; *Sult1a1*, sulfotransferase family 1A, phenol-preferring, member 1; *Timm10b*, translocase of inner mitochondrial membrane 10B; *Srpk2*, serine/arginine-rich protein specific kinase 2; *Mir27b*, microRNA 27b; *Pcgf5*, polycomb group ring finger 5; *Pam*, peptidylglycine alpha-amidating monooxygenase; *Ndel1*, nudE neurodevelopment protein 1 like 1; *Ghdc*, GH3 domain containing; *Mark4*, MAP/microtubule affinity regulating kinase 4; *Med16*, mediator complex subunit 16; *Med19*, mediator complex subunit 19; *Kcnh2*, potassium voltage-gated channel, subfamily H (eag-related), member 2; *Piezo1*, piezo-type mechanosensitive ion channel component 1; *Bak1*, BCL2-antagonist/killer 1; *Aqr*, aquarius; *Exoc5*, exocyst complex component 5; *Tmbim1*, transmembrane BAX inhibitor motif containing 1.

Gene	log2 fold change	Adjusted p-value	Gene	log2 fold change	Adjusted p-value
Ptpn23	38.02	$3.25 \cdot 10^{-18}$	*Pprc1*	−39.91	$8.25 \cdot 10^{-20}$
Cdpf1	31.03	$1.30 \cdot 10^{-16}$	*Nr2c2ap*	−38.85	$6.13 \cdot 10^{-19}$
Mir22	33.55	$9.39 \cdot 10^{-16}$	*Akt1*	−31.16	$3.91 \cdot 10^{-18}$
Mir1949	34.44	$4.13 \cdot 10^{-15}$	*Mto1*	−34.6	$3.31 \cdot 10^{-15}$
Frmd4b	33.81	$1.36 \cdot 10^{-14}$	*Nras*	−32.07	$4.13 \cdot 10^{-15}$
Snf8	30.14	$2.35 \cdot 10^{-14}$	*Nadk2*	−33.92	$1.13 \cdot 10^{-14}$
Htra2	31.28	$3.74 \cdot 10^{-12}$	*Cxcl9*	−32.82	$8.80 \cdot 10^{-14}$
Cerk	25.99	$1.46 \cdot 10^{-10}$	*Zmat5*	−28.98	$1.36 \cdot 10^{-13}$
Ttc33	26.66	$5.00 \cdot 10^{-10}$	*Gbp9*	−32.25	$2.63 \cdot 10^{-13}$
Net1	27.51	$1.53 \cdot 10^{-9}$	*Scyl1*	−31.18	$2.21 \cdot 10^{-12}$
Sult1a1	25.0	$2.12 \cdot 10^{-9}$	*Hypk*	−29.95	$2.15 \cdot 10^{-11}$
Pam	26.51	$8.44 \cdot 10^{-9}$	*Guk1*	−28.29	$4.45 \cdot 10^{-10}$
Ndel1	26.14	$1.47 \cdot 10^{-8}$	*Mrrf*	−23.83	$1.34 \cdot 10^{-9}$
Ghdc	25.74	$2.60 \cdot 10^{-8}$	*Tut1*	−26.96	$4.03 \cdot 10^{-9}$
Mir26b	25.47	$3.86 \cdot 10^{-8}$	*Izumo4*	−26.55	$8.14 \cdot 10^{-9}$
Smg9	7.97	$7.91 \cdot 10^{-8}$	*Sppl3*	−25.96	$1.11 \cdot 10^{-8}$
Atmin	9.19	$1.00 \cdot 10^{-7}$	*Slc39a9*	−26.31	$1.13 \cdot 10^{-8}$
Rtl8c	10.11	$1.03 \cdot 10^{-7}$	*E4f1*	−26.08	$1.57 \cdot 10^{-8}$
Med16	24.68	$1.19 \cdot 10^{-7}$	*Adprm*	−21.6	$1.70 \cdot 10^{-8}$
Exoc5	24.2	$2.41 \cdot 10^{-7}$	*Cracr2b*	−25.9	$2.05 \cdot 10^{-8}$

Table S2. List of genes that exhibit significant up-regulation (left) or down-regulation (right) between adolescence (15 weeks) and adulthood (8 months). Each table includes the top 20 up- or down-regulated genes with the lowest p-values calculated by Wald test between the 15 week and 8 month age groups, sorted by adjusted p-value. Benjamini-Hochberg multiple testing correction was used to adjust p-values. The p-values provide an indication of the magnitude of expression differences for each gene between the two time points. DESeq2's shrunken log2 fold change (LFC) for each gene is also listed.

Upregulated transcripts: *Ptpn23*, protein tyrosine phosphatase, non-receptor type 23; *Cdpf1*, cysteine rich, DPF motif domain containing 1; *Mir22*, microRNA 22; *Mir1949*, microRNA 1949; *Frmd4b*, FERM domain containing 4B; *Snf8*, SNF8, ESCRT-II complex subunit, homolog (S. cerevisiae); *Htra2*, HtrA serine peptidase 2; *Cerk*, ceramide kinase; *Ttc33*, tetratricopeptide repeat domain 33; *Net1*, neuroepithelial cell transforming gene 1; *Sult1a1*, sulfotransferase family 1A, phenol-preferring, member 1; *Pam*, peptidylglycine alpha-amidating monooxygenase; *Ndel1*, nudE neurodevelopment protein 1 like 1; *Ghdc*, GH3 domain containing; *Mir26b*, microRNA 26b; *Smg9*, smg-9 homolog, nonsense mediated mRNA decay factor (C. elegans); *Atmin*, ATM interactor; *Rtl8c*, retrotransposon Gag like 8C; *Med16*, mediator complex subunit 16; *Exoc5*, exocyst complex component 5.

Downregulated transcripts: *Pprc1*, peroxisome proliferative activated receptor, gamma, coactivator-related 1; *Nr2c2ap*, nuclear receptor 2C2-associated protein; *Akt1*, thymoma viral proto-oncogene 1; *Mto1*, mitochondrial tRNA translation optimization 1; *Nras*, neuroblastoma ras oncogene; *Nadk2*, NAD kinase 2, mitochondrial; *Cxcl9*, chemokine (C-X-C motif) ligand 9; *Zmat5*, zinc finger, matrin type 5; *Gbp9*, guanylate-binding protein 9; *Scyl1*, SCY1-like 1 (S. cerevisiae); *Hypk*, huntingtin interacting protein K; *Guk1*, guanylate kinase 1; *Mrrf*, mitochondrial ribosome recycling factor; *Tut1*, terminal uridylyl transferase 1, U6 snRNA-specific; *Izumo4*, IZUMO family member 4; *Sppl3*, signal peptide peptidase 3; *Slc39a9*, solute carrier family 39 (zinc transporter), member 9; *E4f1*, E4F transcription factor 1; *Adprm*, ADP-ribose/CDP-alcohol diphosphatase, manganese dependent; *Cracr2b*, calcium release activated channel regulator 2B.

Gene	log2 fold change	Adjusted p-value
Akt1	33.38	$2.34 \cdot 10^{-18}$
Pprc1	39.06	$5.55 \cdot 10^{-17}$
Nras	32.99	$6.00 \cdot 10^{-14}$
Gbp9	33.19	$3.23 \cdot 10^{-12}$
Nr2c2ap	32.81	$6.67 \cdot 10^{-12}$
Mto1	32.23	$1.50 \cdot 10^{-11}$
Zmat5	28.32	$2.56 \cdot 10^{-11}$
Nadk2	31.81	$2.69 \cdot 10^{-11}$
Cxcl9	31.15	$7.46 \cdot 10^{-11}$
Scyl1	31.06	$8.69 \cdot 10^{-11}$
Izumo4	28.86	$2.16 \cdot 10^{-9}$
Mrrf	24.52	$3.68 \cdot 10^{-9}$
Selenok	10.1	$2.86 \cdot 10^{-8}$
Nup37	24.27	$4.77 \cdot 10^{-8}$
E4f1	25.8	$1.56 \cdot 10^{-7}$
Adprm	21.35	$1.58 \cdot 10^{-7}$
Sppl3	25.39	$1.59 \cdot 10^{-7}$
Prelid1	23.72	$1.72 \cdot 10^{-7}$
Usp16	25.32	$2.74 \cdot 10^{-7}$
Vsig2	23.11	$3.43 \cdot 10^{-7}$

Gene	log2 fold change	Adjusted p-value
Mcm7	-33.58	$3.38 \cdot 10^{-28}$
Fkbp10	-39.61	$8.94 \cdot 10^{-25}$
Mageb2	-26.19	$3.93 \cdot 10^{-23}$
Efnb1	-26.4	$9.29 \cdot 10^{-22}$
Pdzd11	-24.63	$7.65 \cdot 10^{-19}$
Mageb1	-24.44	$2.53 \cdot 10^{-17}$
Yipf6	-24.3	$5.46 \cdot 10^{-17}$
Rn7s1	-24.39	$1.00 \cdot 10^{-15}$
Stard8	-24.24	$1.59 \cdot 10^{-15}$
Mir223	-23.8	$2.10 \cdot 10^{-14}$
Las1l	-22.46	$2.16 \cdot 10^{-14}$
Dnmt1	-21.69	$1.87 \cdot 10^{-13}$
Hba-a2	-33.89	$1.89 \cdot 10^{-13}$
Rpl17-ps8	-21.32	$9.43 \cdot 10^{-13}$
Hdac7	-22.82	$1.52 \cdot 10^{-12}$
Foxo4	-23.38	$2.99 \cdot 10^{-12}$
Ddx3y	-33.45	$3.48 \cdot 10^{-12}$
Tspan9	-32.93	$7.99 \cdot 10^{-12}$
Xkrx	-28.93	$8.26 \cdot 10^{-12}$
Gprasp2	-22.59	$2.69 \cdot 10^{-11}$

Table S3. List of genes that exhibit significant up-regulation (left) or down-regulation (right) in old age. Each table includes the top 20 up- and down-regulated genes with the lowest p-values calculated by Wald test between the 8 month and 22 month age groups, sorted by adjusted p-value. The Benjamini-Hochberg multiple testing correction was used to adjust p-values. The p-values provide an indication of the magnitude of expression differences for each gene between the two time points. DESeq2's shrunken log2 fold change (LFC) for each gene is also listed.

Upregulated transcripts: *Akt1*, thymoma viral proto-oncogene 1; *Pprc1*, peroxisome proliferative activated receptor, gamma, coactivator-related 1; *Nras*, neuroblastoma ras oncogene; *Gbp9*, guanylate-binding protein 9; *Nr2c2ap*, nuclear receptor 2C2-associated protein; *Mto1*, mitochondrial tRNA translation optimization 1; *Zmat5*, zinc finger, matrin type 5; *Nadk2*, NAD kinase 2, mitochondrial; *Cxcl9*, chemokine (C-X-C motif) ligand 9; *Scyl1*, SCY1-like 1 (S. cerevisiae); *Izumo4*, IZUMO family member 4; *Mrrf*, mitochondrial ribosome recycling factor; *Selenok*, selenoprotein K; *Nup37*, nucleoporin 37; *E4f1*, E4F transcription factor 1; *Adprm*, ADP-ribose/CDP-alcohol diphosphatase, manganese dependent; *Sppl3*, signal peptide peptidase 3; *Prelid1*, PRELI domain containing 1; *Usp16*, ubiquitin specific peptidase 16; *Vsig2*, V-set and immunoglobulin domain containing 2.

Downregulated transcripts: *Mcm7*, minichromosome maintenance complex component 7; *Fkbp10*, FK506 binding protein 10; *Mageb2*, melanoma antigen, family B, 2; *Efnb1*, ephrin B1; *Pdzd11*, PDZ domain containing 11; *Mageb1*, melanoma antigen, family B, 1; *Yipf6*, Yip1 domain family, member 6; *Rn7s1*, 7S RNA 1; *Stard8*, START domain containing 8; *Mir223*, microRNA 223; *Las1l*, LAS1-like (S. cerevisiae); *Dnmt1*, DNA methyltransferase (cytosine-5) 1; *Hba-a2*, hemoglobin alpha, adult chain 2; *Rpl17-ps8*, ribosomal protein L17, pseudogene 8; *Hdac7*, histone deacetylase 7; *Foxo4*, forkhead box O4; *Ddx3y*, DEAD (Asp-Glu-Ala-Asp) box polypeptide 3, Y-linked; *Tspan9*, tetraspanin 9; *Xkrx*, X-linked Kx blood group related, X-linked; *Gprasp2*, G protein-coupled receptor associated sorting protein 2.

Ontology	Description	Adjusted p-value	DE genes
BP	protein localization to cell periphery	0.029	*Exoc5*, *Akt1*, *Cltc*, *Tmbim1*, *Ank1*, *Rock1*, *Mrap*, *Pid1*, *Camk2d*, *Snap23*, *Ppil2*, *Lyplal1*, *Mal*, *Lgals3*, *Gsk3b*, *Ankrd9*, *Myadm*
BP	negative regulation of protein localization to plasma membrane	0.029	*Cltc*, *Tmbim1*, *Mrap*, *Pid1*, *Lyplal1*
BP	negative regulation of protein localization to cell periphery	0.030	*Cltc*, *Tmbim1*, *Mrap*, *Pid1*, *Lyplal1*
BP	respiratory burst	0.030	*Cybc1*, *Cyba*, *Insr*, *Selenok*, *Bcr*
BP	negative regulation of cellular protein localization	0.030	*Cltc*, *Tmbim1*, *Fbxo4*, *Tax1bp3*, *Mrap*, *Flcn*, *Pid1*, *Lyplal1*, *Gsk3b*
CC	ubiquitin ligase complex	0.0005	*Pcgf5*, *Glmn*, *Fbxo4*, *Anapc5*, *Dcaf1*, *Fbxw5*, *Dcun1d5*, *Traf2*, *Ube4b*, *Spsb2*, *Cand1*, *Ube2d2a*, *Med11*, *Depdc5*, *Rnf7*, *Dcaf12l1*, *Mkln1*
CC	Cul4-RING E3 ubiquitin ligase complex	0.018	*Glmn*, *Dcaf1*, *Fbxw5*, *Rnf7*, *Dcaf12l1*
CC	cullin-RING ubiquitin ligase complex	0.018	*Glmn*, *Fbxo4*, *Anapc5*, *Dcaf1*, *Fbxw5*, *Spsb2*, *Cand1*, *Depdc5*, *Rnf7*, *Dcaf12l1*
CC	vesicle coat	0.019	*Cltc*, *Scyl1*, *Ap1g1*, *Cltb*, *Tmed3*, *Ap2s1*
CC	hemidesmosome	0.022	*Plec*, *Dst*, *Itga6*
MF	protein serine/threonine kinase activity	0.002	*Map3k4*, *Srpk2*, *Mark4*, *Akt1*, *Nme2*, *Dcaf1*, *Rock1*, *Sik3*, *Bckdk*, *Grk3*, *Eif2ak1*, *Bcr*, *Pak4*, *Camk2d*, *Pdk2*, *Cpne3*, *Mapkapk3*, *Gsk3b*, *Csnk2b*, *Haspin*, *Wnk1*
MF	14-3-3 protein binding	0.015	*Srpk2*, *Akt1*, *Ppp1r12a*, *Dab2ip*, *Hdac7*
MF	ubiquitin protein ligase activity	0.026	*Fbxo4*, *Anapc5*, *Chfr*, *Traf6*, *D7Ertd443e*, *Ube4b*, *Nosip*, *Siah2*, *Mycbp2*, *Med11*, *Ppil2*, *Rnf7*
MF	ubiquitin-like protein ligase activity	0.026	*Fbxo4*, *Anapc5*, *Chfr*, *Traf6*, *D7Ertd443e*, *Ube4b*, *Nosip*, *Siah2*, *Mycbp2*, *Med11*, *Ppil2*, *Rnf7*
MF	ubiquitin-protein transferase activity	0.043	*Fbxo4*, *Anapc5*, *Chfr*, *Traf6*, *D7Ertd443e*, *Traf2*, *Ube4b*, *Nosip*, *Trim8*, *Siah2*, *Ube2d2a*, *Mycbp2*, *Med11*, *Ppil2*, *Rnf7*

Table S4. List of gene ontologies (GO) that exhibit significant enrichment between the 4 week and 15 week age groups (adjusted p-value < 0.05). GO enrichment analysis was performed using the R package clusterProfiler (Yu et al., 2012). The left-most column indicates the type of subontology to which each GO belongs: BP (biological process), MF (molecular function), or CC (cellular compartment). For each subontology, the five GOs with the lowest p-values are displayed. The DE genes listed in the right-most column are the genes belonging to each ontology that were identified as differentially-expressed by the Wald test (adjusted p-value < 0.05).

Ontology	Description	Adjusted p-value	DE genes
BP	regulation of endoplasmic reticulum unfolded protein response	0.015	*Bak1*, *Xbp1*, *Tmem33*, *Ptpn1*, *Nck1*
BP	mitochondrion organization	0.015	*Akt1*, *Htra2*, *Rhot2*, *Bak1*, *Prelid1*, *P2rx7*, *Pid1*, *Ndufb7*, *Ndufs5*, *Bcs1l*, *Myc*, *Sdhaf2*, *Cox17*, *Ndufaf8*, *Smurf1*, *Ndufa3*, *Dmac2*, *Col4a3bp*, *Tspo*, *Hspa4*
BP	mitochondrial respiratory chain complex assembly	0.015	*Ndufb7*, *Ndufs5*, *Bcs1l*, *Sdhaf2*, *Cox17*, *Ndufaf8*, *Ndufa3*, *Dmac2*
BP	NADH dehydrogenase complex assembly	0.027	*Ndufb7*, *Ndufs5*, *Bcs1l*, *Ndufaf8*, *Ndufa3*, *Dmac2*
BP	mitochondrial respiratory chain complex I assembly	0.027	*Ndufb7*, *Ndufs5*, *Bcs1l*, *Ndufaf8*, *Ndufa3*, *Dmac2*
CC	mitochondrial intermembrane space	0.005	*Htra2*, *Timm10b*, *Prelid1*, *Gatm*, *Ndufb7*, *Cox17*, *Micu1*, *Hax1*
CC	organelle envelope lumen	0.008	*Htra2*, *Timm10b*, *Prelid1*, *Gatm*, *Ndufb7*, *Cox17*, *Micu1*, *Hax1*
CC	organelle inner membrane	0.028	*Timm10b*, *Rhot2*, *Ndufb4*, *Noa1*, *Cox15*, *Lgals3*, *Cox5a*, *P2rx7*, *Gatm*, *Dpy19l3*, *Ndufb7*, *Slc25a1*, *Ndufs5*, *Bcs1l*, *Micu1*, *Ndufa3*, *Dmac2*, *Itpr1*
CC	oligosaccharyltransferase complex	0.028	*Dad1*, *Krtcap2*, *Ddost*
CC	mitochondrial membrane part	0.028	*Timm10b*, *Rhot2*, *Ndufb4*, *Noa1*, *Bak1*, *Cox5a*, *Ndufb7*, *Ndufs5*, *Micu1*, *Ndufa3*, *Dmac2*
MF	protein C-terminus binding	0.004	*Snf8*, *Tax1bp3*, *Ercc1*, *Ap1g1*, *Dapk3*, *Grip2*, *Ube2i*, *Epb41*, *Procr*, *Pex16*, *Mif4gd*, *Atp1b1*, *Itpr1*, *Ptk2b*
MF	ubiquitin protein ligase binding	0.039	*Nploc4*, *Kcnh2*, *Xbp1*, *Glmn*, *Ube2k*, *Cacul1*, *Psmd1*, *Myc*, *Traf6*, *Vcl*, *Actg1*, *Ptk2b*, *Slf1*, *Cul9*
MF	ubiquitin-like protein ligase binding	0.048	*Nploc4*, *Kcnh2*, *Xbp1*, *Glmn*, *Ube2k*, *Cacul1*, *Psmd1*, *Myc*, *Traf6*, *Vcl*, *Actg1*, *Ptk2b*, *Slf1*, *Cul9*

Table S5. List of gene ontologies (GO) that exhibit significant enrichment between the 15 week and 8 month age groups (adjusted p-value < 0.05). GO enrichment analysis was performed using the R package clusterProfiler(Yu et al., 2012). The left-most column indicates the type of subontology to which each GO belongs: BP (biological process), MF (molecular function), or CC (cellular compartment). For each subontology, the five GOs with the lowest p-values are displayed. The DE genes listed in the right-most column are the genes belonging to each ontology that were identified as differentially-expressed by the Wald test (adjusted p-value < 0.05).
